# Suicidality during neuromodulation in the elderly depressed: study design

**DOI:** 10.1192/j.eurpsy.2022.1902

**Published:** 2022-09-01

**Authors:** A. Van Daele, D. Zeeuws, C. Baeken

**Affiliations:** 1 Vrije Universiteit Brussel, Faculty Of Medicine And Pharmacy, Jette, Belgium; 2 University Hospital Brussels, Department Of Psychiatry, Jette, Belgium; 3 Vrije Universiteit Brussel, Center For Neurosciences (c4n), Jette, Belgium; 4 Ghent University, Psychiatry, Gent, Belgium; 5 Eindhoven University of Technology, Department Of Electrical Engineering, Eindhoven, Netherlands

## Abstract

**Introduction:**

Late life depression is a major global health issue, with an estimated 7% of older adults suffering from this mental disorder. Depression is one of the most important predictors for suicide in the elderly. However, it is often difficult to recognize and manage, making treatment-resistance a common occurrence. Treatment-resistant depression itself is also a known risk factor for suicide. Recently, non-invasive neuromodulation techniques have been used as a new treatment for depression and suicidality with promising results.

**Objectives:**

This study aims to investigate the effect of adTMS (accelerated deep Transcranial Magnetic Stimulation) and tDCS (transcranial Direct Current Stimulation) on the suicidality of elderly, therapy-resistant depressed patients. The hypothesis is that suicidal ideation and risk of suicide will decrease after a treatment with adTMS and tDCS.

**Methods:**

In this randomized double-blinded sham-controlled clinical trial, geriatric therapy-resistant depressive patients will receive adTMS treatment (See: Figure 1). Suicidality will be assessed before and after the active or sham treatment, through the Columbia Suicide Severity Rating Scale (C-SSRS) and Beck Scale for Suicide Ideation (BSI). After one week of rest, all patients will receive an at-home tDCS treatment for 3 weeks. Likewise, the suicide risk will be estimated before and after the tDCS. During the screening period, the severity of the patients’ depressive symptoms will be determined by using the 17-item Hamilton Depression Rating Scale (HDRS-17). In total, the trial will last for 5 weeks, and suicidality will be examined at five different time points (during screening, at T0, T1, T2 and T3).

**Figure:**
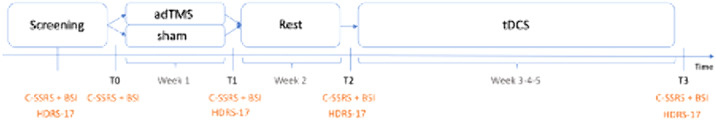

**Results:**

Not applicable

**Conclusions:**

Not applicable

**Disclosure:**

No significant relationships.

